# Estimating HIV pre-exposure prophylaxis need and impact in Malawi, Mozambique and Zambia: A geospatial and risk-based analysis

**DOI:** 10.1371/journal.pmed.1003482

**Published:** 2021-01-11

**Authors:** Dominik Stelzle, Peter Godfrey-Faussett, Chuan Jia, Obreniokibo Amiesimaka, Mary Mahy, Delivette Castor, Ioannis Hodges-Mameletzis, Lastone Chitembo, Rachel Baggaley, Shona Dalal

**Affiliations:** 1 Center for Global Health, Department of Neurology, Technical University, Munich, Germany; 2 Chair of Epidemiology, Faculty of Sport and Health Sciences, Technical University, Munich, Germany; 3 Strategic Information Department, UNAIDS, Geneva, Switzerland; 4 Clinical Research Department, London School of Hygiene and Tropical Medicine, London, United Kingdom; 5 Global HIV, Hepatitis and STIs Programmes, World Health Organization, Geneva, Switzerland; 6 Department of Medicine, Division of Infectious Diseases, Columbia University Irving Medical Center, New York, New York, United States of America; 7 World Health Organization, Lusaka, Zambia; Makerere University University, UGANDA

## Abstract

**Background:**

Pre-exposure prophylaxis (PrEP), a WHO-recommended HIV prevention method for people at high risk for acquiring HIV, is being increasingly implemented in many countries. Setting programmatic targets, particularly in generalised epidemics, could incorporate estimates of the size of the population likely to be eligible for PrEP using incidence-based thresholds. We estimated the proportion of men and women who would be eligible for PrEP and the number of HIV infections that could be averted in Malawi, Mozambique, and Zambia using prioritisation based on age, sex, geography, and markers of risk.

**Methods and findings:**

We analysed the latest nationally representative Demographic and Health Surveys (DHS) of Malawi, Mozambique, and Zambia to determine the proportion of adults who report behavioural markers of risk for HIV infection. We used prevalence ratios (PRs) to quantify the association of these factors with HIV status. Using a multiplier method, we combined these proportions with the number of new HIV infections by district, derived from district-level modelled HIV estimates. Based on these numbers, different scenarios were analysed for the minimum number of person-years on PrEP needed to prevent 1 HIV infection (NNP).

An estimated total of 38,000, 108,000, and 46,000 new infections occurred in Malawi, Mozambique, and Zambia in 2016, corresponding with incidence rates of 0.43, 0.63, and 0.57 per 100 person-years. In these countries, 9%–20% of new infections occurred among people with a sexually transmitted infection (STI) in the past 12 months and 40%–42% among people with either an STI or a non-regular sexual partner (NP) in the past 12 months (STINP). The models estimate that around 50% of new infections occurred in districts with incidence rates ≥1.0% in Mozambique and Zambia and ≥0.5% in Malawi. In Malawi, Mozambique, and Zambia, 35.1%, 21.9%, and 12.5% of the population live in these high-incidence districts. In the most parsimonious scenario, if women aged 15–34 years and men 20–34 years with an STI in the past 12 months living in high-incidence districts were to take PrEP, it would take a minimum of 65.8 person-years on PrEP to avert 1 HIV infection per year in Malawi, 35.2 in Mozambique, and 16.4 in Zambia. Our findings suggest that 3,300, 5,200, and 1,700 new infections could be averted per year in the 3 countries, respectively. Limitations of our study are that these values are based on modelled estimates of HIV incidence and self-reported behavioural risk factors from national surveys.

**Conclusions:**

A large proportion of new HIV infections in these 3 African countries were estimated to occur among people who had either an STI or an NP in the past year, providing a straightforward means to set PrEP targets. Greater prioritisation of PrEP by district, sex, age, and behavioural risk factors resulted in lower NNPs thereby increasing PrEP cost-effectiveness, but also diminished the overall impact on reducing new infections

## Abstract 2

Dominik Stelzle and co-workers estimate impact of antiretroviral pre-exposure prophylaxis use on HIV infections in 3 African countries.

## Author summary

**Why was this study done?:**

- WHO recommends oral pre-exposure prophylaxis (PrEP) for people at substantial risk of HIV to prevent HIV acquisition, currently defined as 3% or higher per annum.
- Prioritising those at “substantial risk” increases the cost-effectiveness of PrEP.
- Setting programmatic targets to estimate the number of people who are at substantial risk and who could benefit from PrEP is challenging.
- This study was done to provide a straightforward approach, with some country examples, to estimate PrEP need and to help prioritise the offer of PrEP in settings with high HIV prevalence.

**What did the researchers do and find?:**

- We used modelled subnational age- and sex-specific HIV incidence estimates with population survey data from 3 countries to generate estimates of the increase in risk for people with behaviours that can be reported programmatically.
- Applying both these estimates together enabled us to calculate how many people fall into different broad categories of substantial HIV risk and therefore the sizes of potential populations that could benefit from PrEP.
- It also allows estimating the likely impact that PrEP could have on reducing new HIV infections if prioritised in this way.
- We found that the most focused approach of offering PrEP to women aged 15–34 years and men 20–34 years, with a sexually transmitted infection (STI) in the past 12 months in high-incidence districts, lowered the number of person-years on PrEP needed to avert 1 HIV infection. A less prioritised approach, encouraging broader use of PrEP, would have a greater impact on population-level reduction of HIV infections, but will cost more.

**What do these findings mean?:**

- For comparatively more expensive prevention interventions like PrEP, prioritising factors such as geography, age, sex, and sexual behaviour allows subgroups with high average HIV incidence to be identified.
- As PrEP is scaled up, policy makers and funders will have to make careful decisions that balance costs, impact, demand, and the basic rights of individuals to access highly effective prevention tools.

## Introduction

In 2019, there were an estimated 38.0 million people living with HIV (PLHIV) and 1.7 million new HIV infections worldwide, far distant from the Joint United Nations Programme on HIV/AIDS (UNAIDS) global target of fewer than 500,000 new infections by 2020 [1,2]. Eastern and southern Africa alone contributed about 43% of global new HIV infections and 53% of worldwide prevalent HIV infections in 2019 [2]. Women aged 15 to 24 years old who comprise just 10% of the population in southern and eastern Africa, contributed 26% of new infections in 2019 [3]. Reducing new infections will require strengthening and scaling up a combination of effective HIV prevention strategies, identifying people with undiagnosed HIV, and expanding treatment coverage.

Pre-exposure prophylaxis (PrEP) is an effective HIV prevention method that entails the use of oral daily tenofovir disoproxil fumarate (TDF)-containing antiretrovirals by HIV–negative individuals at substantial risk to prevent HIV acquisition [4,5]. PrEP has been shown to reduce the risk of contracting HIV in trials by up to 97% in men and transgender women both of whom have sex with men [6]. PrEP has also been shown in trials to be highly effective in reducing HIV acquisition in heterosexual men and women [7]. WHO and UNAIDS estimated that PrEP may be cost-effective especially when HIV incidence in subpopulations exceeds 3 per 100 person-years in the absence of PrEP and referred to this level of incidence as “substantial” risk [8]. Others have estimated this incidence threshold to be higher and influenced by overall prevention expenditure [9], and other countries such as the United States have moved ahead with lower thresholds [10]. These thresholds need to be interpreted in the context of risks that may vary considerably from month to month [11]. High levels of HIV incidence continue to be reported in the general population within certain communities in eastern and southern Africa where this threshold may be relevant [8,12,13]. Adherence to the prescribed PrEP regimen is critical for effectiveness [14,15]. Although the cost of PrEP is lower than lifelong antiretroviral therapy (ART), it is a comparatively more expensive preventive intervention compared to male and female condoms or voluntary medical male circumcision.

Establishing the size of the population who are likely to be at “substantial” risk in general population settings (outside of key populations where HIV incidence remains higher than the overall population) could assist programmes in setting annual targets for PrEP drug procurement and delivery of PrEP services. We estimated the proportion of men and women who would be eligible for PrEP and the number of HIV infections that could be averted in Malawi, Mozambique, and Zambia in different scenarios using prioritisation based on geography, age, sex, and survey markers of HIV risk.

## Methods

We used 2 sources of data to calculate the size of the population at risk of HIV and in need of PrEP using different prioritisation criteria. We analysed the latest Demographic and Health Surveys (DHS) to determine the effect of association between certain behavioural factors and HIV status and the proportion of people who reported these risk factors. We also used the 2016 modelled estimates (HIVE-Map Geospatial Model) for the number of new HIV infections nationally as well as disaggregated HIV incidence by age, sex, and district for each country [16,17]. Three countries—Malawi, Mozambique, and Zambia—were selected for analysis because all 3 had both recent DHS including HIV testing (Malawi (DHS 2015/2016), Mozambique (AIDS Indicator Surveys (AIS) 2015), Zambia (DHS 2013/2014)) and 2016 district-level HIV incidence estimates (HIVE estimates) available.

### Demographic and health surveys

DHS are nationally representative household surveys that provide data for a wide range of indicators in the areas of population, health, and nutrition. In some DHS, HIV testing is included. AIS are a special subtype of DHS focusing on indicators for the effective monitoring of national HIV/AIDS programs [18].

We chose known risk factors for incident HIV infections that were available in DHS datasets. These were reporting symptoms of a sexually transmitted infection (STI) in the past 12 months or have non-regular sexual partners (NPs) in the past 12 months. We categorised a response of “yes” to any of the following 3 questions as having had an STI in the past 12 months: “Have you had any STI in last 12 months?,” “Have you had a genital sore/ulcer in last 12 months?,” and “Have you had a genital discharge in last 12 months?.” People who reported having at least 1 sexual partner who was not a spouse or cohabiting partner were considered to have had an NP. Out of these questions, we created 2 risk variables: (A) people who reported an STI in the past 12 months (STI); and (B) people who reported either an STI or at least 1 NP in the past 12 months (sexually transmitted infection or non-regular partners (STINP)).

To determine the association between the behavioural factors and HIV prevalence, we calculated prevalence ratios (PRs) by 5-year age groups and sex (PR_asr_). For this analysis, we combined the datasets of all 3 countries in order to increase the number of cases in each group. If less than 50 weighted cases occurred in a specific age group, this age group was merged with the next older one. For the variable STI in the past 12 months, the age groups 15 to 19 and 20 to 24 years were combined for women, and the age groups 15 to 19, 20 to 24, and 25 to 29 years were combined for men; for the variables STINP (STI or NP) in the past 12 months, for men, the age groups 15 to 19 years and 20 to 24 years were combined (S1A and S1B Tables).

### Geospatial incidence estimates

The HIVE-Map Geospatial Model estimates the distribution of new HIV infections, PLHIV, and ART coverage at district level and by age and sex [16,17]. The model uses facility level prevalence among pregnant women attending antenatal care, facility-level number of people on antiretroviral treatment, cluster-level survey data on prevalence among adults, 5 × 5 km demographic data from AfriPop, and a suite of 5 ×5 km resolution geospatial covariates that are predictive of spatial variation in HIV risk. Pixel-level predictions are scaled within each country or administrative unit to ensure consistency with national Spectrum estimates created in 2016 and used for national planning. District-level estimates of PLHIV, new infections, and ART coverage were produced by sex and age group for 10 countries in late 2017 and early 2018 [16,17].

### HIV incidence calculation and number of person-years on PrEP needed to prevent one HIV infection (NNP)

We assumed that all new HIV infections among adults occurred among sexually active people, since it is the predominant mode of transmission in African countries [19]. Sexually active was defined as people reporting having had sex in the past 12 months.

For the calculation of the number of people at risk of HIV infection by age, sex, and district (R_asrd_), the proportion of people with STI or STINP in the past 12 months by age and sex (P_asr_) was multiplied by the number of people by age, sex, and district (N_asd_) in each country. The following formula was used:

$$R_{asrd}=P_{asr}*N_{asd}.$$

We then combined the PRs and the number of people with the risk factor using the following formula:

$$I_{asrd}=I_{asd}*\frac{\left(R_{asrd}*{PR}_{asr}\right)}{\left(Q_{asrd}+(R_{asrd}*{PR}_{asr})\right)}$$

I_asrd_: Incident HIV infections in the group with the risk factor, by age, sex, and district

I_asd_: Incident HIV infections in the country, by age, sex, and district

R_asrd_: Number of people with the risk factor, by age, sex, and district

Q_asrd_: Number of people without the risk factor, by age, sex, and district

PR_asr_: Prevalence ratio by age, sex, and risk factor

Based on I_asrd_, we analysed different scenarios for HIV incidence as well as the number of person-years on PrEP needed to prevent 1 HIV infection (NNP). We assumed 100% drug efficacy. In a more realistic setting, allowing for factors such as low coverage, low uptake, and high discontinuation that occur during service delivery, we assumed a PrEP effectiveness of 50% with an uncertainty interval from 25% to 75% [15]. The scenarios for PrEP provision were the following: all adults (scenario 1), by age group (scenario 2), by district HIV incidence (scenario 3), and by age, sex, and district HIV incidence (scenario 4). For each scenario, data are additionally presented for the subset of people with STINP and the people with STI in the past 12 months.

For the scenarios that included HIV incidence by district, cutoffs were chosen to define high-incidence districts as those where approximately 50% of all new infections occurred, middle incidence districts where approximately 25% of new infections occurred, and low incidence districts had <25% of new infections. The cutoffs were then rounded to the nearest 10th of a percent. The district incidence rate cutoffs were 1% and 0.5% for Zambia and Mozambique and 0.5% and 0.3% for Malawi.

All analyses were prespecified and were run using Microsoft Excel and R version 3.5.1. We did not have a protocol or prespecified analysis plan for this study. Reporting followed the RECORD checklist (S2 Table).

### Ethical approval

All the utilised DHS and AIS datasets are publicly available. The DHS programme data are de-identified before made available to the public. No data on individual participants was presented. Therefore, this work did not require ethical approval.

## Results

In the year 2016, an estimated total number of 38,000 adults (aged 15 years and older) were newly infected with HIV in Malawi, 108,000 in Mozambique, and 46,000 in Zambia. HIV incidence derived from the HIVE models was 0.41% in Malawi, 0.56% in Mozambique, and 0.54% in Zambia. In all 3 countries, women were more likely to get infected with HIV, accounting for 56%, 58%, and 57% of all new infections in Malawi, Mozambique, and Zambia. HIV prevalence ranged from 7.9% (95% CI 6.2 to 8.0) among men in Malawi to 14.4% (95% CI 13.7 to 17.2) among women in Zambia. In all 3 countries, HIV prevalence was higher among women than men.

The prevalence of reported STIs across all 3 countries ranged from 3.8% to 13.6% and was highest in Malawi (Table 1). The proportion of people who reported either an STI or had NPs in the past 12 months was similar across the 3 countries (range 23.5 to 27.5%) and was higher among men than women. In total numbers, in Malawi, Mozambique, and Zambia, 2.6, 5.3, and 2.2 million people were categorised as STINP in the past 12 months. Of those, 1.1, 1.2, and 0.4 million people only had an STI.

**Table 1:** Number of new HIV infections, incidence, prevalence, and proportion of people with an STI or NP in the past 12 months in Malawi, Mozambique, and Zambia.

Country		Population 15 years and older (in million) 2016	New HIV infections 2016	Proportion of new infections	HIV incidence (%)*	HIV prevalence*	STINP in the past 12 months	NP in the past 12 months	STI in the past 12 months
Malawi	**All**	**9.9**	**38,000**		**0.43**	**9.8%**	**26.2%**	**18.2%**	**11.3%**
	Females	5.1	21,000	55%	0.49	11.7%	21.9%	10.0%	13.6%
	Males	4.8	17,000	45%	0.37	7.9%	31.2%	26.4%	8.6%
Mozambique	**All**	**19.3**	**108,000**		**0.63**	**10.7%**	**27.5%**	**25.5%**	**6.3%**
	Females	9.9	63,000	58%	0.72	12.5%	20.8%	16.4%	6.2%
	Males	9.3	46,000	43%	0.54	8.8%	37.6%	34.6%	6.5%
Zambia	**All**	**9.2**	**46,000**		**0.57**	**12.2%**	**23.5%**	**21.3%**	**4.3%**
	Females	4.7	27,000	57%	0.67	14.4%	17.4%	14.5%	3.8%
	Males	4.6	20,000	43%	0.48	10.0%	30.4%	28.0%	5.0%

*HIV incidence and prevalence measures were obtained from HIVE modelled estimates.

HIVE, geospatial HIV estimates; NP, non-regular sexual partner; STI, sexually transmitted infection; STINP, sexually transmitted infection or non-regular partners.

In all 3 countries combined, HIV prevalence among women with an STI in the past 12 months was 22.9% compared to 14.6% among women without STI. Among men with STI in the past 12 months, HIV prevalence was 16.6% compared to 10.3% among men without an STI. The PRs showed that women had an increased probability of being infected with HIV if they were in the category STI or STINP regardless of age (PR range for STI: 1.40 to 1.78; PR range for STINP: 1.53 to 2.14). For men, the same was true for those with an STI in the past 12 months (range: 1.64 to 1.85) and for STINP for men aged ≥25 years (PR range 1.31 to 1.75), but the association was reversed in younger men aged 15 to 24 years (S1 and S2 Tables).

### Scenario 1: New HIV infections by sex

In Malawi, Mozambique, and Zambia, 40%, 42%, and 40% of all new infections occurred among people in the category STINP. This corresponds with 15,000, 45,000, and 18,000 new infections. Proportionally, markedly more HIV infections occurred among men than women in the category STINP in the past 12 months in Mozambique (51% among men and 38% among women) (Table 2). For people with an STI alone, a different picture was seen. In Mozambique and Zambia, there was no major difference between men and women, whereas in Malawi, proportionally more infections occurred among women with an STI in the past 12 months (Malawi: 22% versus 16%; Table 2). Using STI in the past 12 months as a marker of risk, HIV incidence exceeded 1% only among women in Mozambique and Zambia.

**Table 2:** New HIV infections and HIV incidence by scenario and priority population.

	Country	Sex	Age group	District incidence (%)	New HIV infections	Proportion of new infections			HIV incidence (%)	HIV incidence (%)		
						Sex in the past 12 months	STINP in the past 12 months			STI in the past 12 months	Sex in the past 12 months	STINP	STI
**Scenario 1**	Malawi	**All**			38,000	100%	40%	20%	0.41	0.53	0.64	0.75
		Females			21,000	100%	41%	22%	0.49	0.66	0.93	0.82
		Males			17,000	100%	39%	16%	0.34	0.43	0.45	0.66
	Mozambique	**All**			108,000	100%	42%	12%	0.56	0.69	0.82	1.08
		Females			63,000	100%	38%	11%	0.63	0.84	1.18	1.20
		Males			46,000	100%	49%	13%	0.49	0.56	0.62	0.97
	Zambia	**All**			46,000	100%	40%	9%	0.54	0.70	0.90	1.05
		Females			26,000	100%	39%	7%	0.66	0.88	1.48	1.27
		Males			20,000	100%	41%	10%	0.43	0.56	0.60	0.90
**Scenario 2**	Malawi	All	15–19		5,900	16%	9%	3%	0.30	0.71	0.54	0.82
			20–24		9,500	25%	11%	5%	0.55	0.69	0.67	0.93
			25–29		8,800	24%	10%	5%	0.59	0.65	0.90	0.93
			30–34		5,600	15%	6%	3%	0.51	0.55	0.82	0.78
			35–39		3,600	10%	3%	2%	0.39	0.43	0.59	0.56
			40–49		3,300	9%	2%	2%	0.27	0.31	0.41	0.44
			50+		NA	NA	NA	NA	NA	NA	NA	NA
			**15–34**		29,800	80%	35%	16%	0.47	0.65	0.69	0.88
		Females	15–19		5,200	25%	13%	5%	0.52	1.16	1.22	1.43
			20–24		5,600	27%	11%	6%	0.65	0.79	1.06	1.01
			25–29		4,400	21%	8%	5%	0.58	0.67	1.01	0.91
			30–34		2,600	12%	5%	3%	0.47	0.54	0.83	0.69
			35–39		1,600	8%	3%	2%	0.35	0.41	0.59	0.48
			40–49		1,500	7%	2%	2%	0.25	0.32	0.44	0.41
			50+		NA	NA	NA	NA	NA	NA	NA	NA
			**15–34**		17,800	85%	36%	19%	0.56	0.78	1.06	0.98
		Males	15–19		800	5%	3%	1%	0.08	0.2	0.13	0.23
			20–24		3,800	23%	11%	4%	0.44	0.59	0.42	0.79
			25–29		4,400	27%	12%	5%	0.59	0.64	0.84	0.95
			30–34		3,000	18%	7%	3%	0.54	0.57	0.82	0.96
			35–39		2,000	12%	3%	2%	0.43	0.45	0.59	0.69
			40–49		1,700	11%	2%	1%	0.30	0.31	0.38	0.51
			50+		800	5%	1%	1%	0.13	0.14	0.17	0.23
			**20–34**		11,200	68%	30%	12%	0.52	0.60	0.62	0.90
	Mozambique	All	15–19		14,000	13%	7%	1%	0.37	0.60	0.54	0.87
			20–24		30,300	28%	13%	4%	0.9	1.00	0.97	1.42
			25–29		24,100	22%	10%	3%	0.88	0.96	1.23	1.39
			30–34		14,700	14%	6%	2%	0.69	0.75	1.07	1.12
			35–39		9,500	9%	3%	1%	0.51	0.57	0.76	0.77
			40–49		10,100	9%	3%	1%	0.40	0.46	0.61	0.70
			50+		5600	5%	1%	0%	0.20	0.29	0.3	0.36
			**15–34**		83,100	77%	36%	10%	0.69	0.84	0.89	1.25
		Females	15–19		9,800	16%	8%	2%	0.52	0.8	0.94	1.11
			20–24		18,600	30%	12%	4%	1.1	1.28	1.71	1.69
			25–29		13,200	21%	8%	3%	0.96	1.13	1.57	1.49
			30–34		7,400	12%	4%	1%	0.69	0.78	1.19	1.03
			35–39		4,700	8%	2%	1%	0.50	0.60	0.84	0.70
			40–49		5,700	9%	3%	1%	0.41	0.52	0.72	0.71
			50+		3,400	5%	1%	0%	0.21	0.4	0.41	0.38
			**15–34**		49,000	78%	32%	10%	0.81	1.02	1.34	1.40
		Males	15–19		4,200	9%	7%	1%	0.22	0.39	0.33	0.58
			20–24		11,800	26%	14%	4%	0.69	0.74	0.63	1.15
			25–29		10,800	24%	14%	4%	0.79	0.81	1.05	1.29
			30–34		7,200	16%	7%	2%	0.68	0.71	0.98	1.2
			35–39		4,800	11%	3%	1%	0.52	0.53	0.7	0.85
			40–49		4,400	10%	2%	1%	0.38	0.4	0.49	0.68
			50+		2,300	5%	1%	1%	0.19	0.21	0.25	0.35
			**20–34**		29,800	65%	34%	10%	0.72	0.76	0.82	1.21
	Zambia	All	15–19		8,800	19%	12%	1%	0.49	1.32	1.02	1.62
			20–24		10,600	23%	10%	2%	0.71	0.98	0.87	1.24
			25–29		8,900	20%	8%	2%	0.71	0.78	1.06	1.20
			30–34		6,600	15%	5%	1%	0.61	0.65	1.01	1.06
			35–39		4,500	10%	3%	1%	0.49	0.53	0.75	0.79
			40–49		4,300	10%	2%	1%	0.36	0.41	0.56	0.66
			50+		NA	NA	NA	NA	NA	NA	NA	NA
			**15–34**		34,900	77%	35%	7%	0.62	0.90	0.98	1.24
		Females	15–19		7,200	28%	17%	2%	0.79	1.94	1.95	2.45
			20–24		6,700	26%	11%	2%	0.89	1.16	1.59	1.61
			25–29		4,700	19%	5%	2%	0.75	0.83	1.44	1.31
			30–34		3,200	12%	3%	1%	0.58	0.64	1.17	0.96
			35–39		2,000	8%	2%	1%	0.43	0.48	0.8	0.66
			40–49		1,700	7%	1%	1%	0.29	0.37	0.61	0.57
			50+		NA	NA	NA	NA	NA	NA	NA	NA
			**15–34**		21,800	86%	36%	6%	0.77	1.08	1.65	1.54
		Males	15–19		1,600	8%	5%	1%	0.18	0.54	0.33	0.62
			20–24		3,900	20%	10%	3%	0.53	0.78	0.54	1.00
			25–29		4,100	21%	11%	2%	0.66	0.74	0.92	1.12
			30–34		3,500	17%	7%	2%	0.63	0.67	0.93	1.13
			35–39		2,500	13%	4%	1%	0.55	0.57	0.72	0.88
			40–49		2,600	13%	3%	1%	0.41	0.44	0.53	0.73
			50+		1,600	8%	1%	1%	0.23	0.25	0.3	0.42
			**20–34**		11,500	58%	28%	7%	0.60	0.73	0.73	1.08
**Scenario 3**	Malawi			**≥1**.**0**	0	-	-	-	-	-	-	-
				0.5–0.99	21,000	56%	23%	11%	0.66	0.86	1.05	1.21
				<0.5	16,000	44%	18%	9%	0.28	0.36	0.44	0.51
	Mozambique			**≥1.0**	53,000	49%	21%	6%	1.26	1.56	1.84	2.40
				0.5–0.99	25,000	23%	10%	3%	0.68	0.83	0.98	1.29
				<0.5	31,000	28%	12%	3%	0.27	0.33	0.40	0.52
	Zambia			**≥1.0**	27,000	59%	24%	5%	2.53	3.30	4.32	4.93
				0.5–0.99	13,000	28%	12%	2%	0.69	0.91	1.18	1.35
				<0.5	5,800	13%	5%	1%	0.11	0.14	0.18	0.21
**Scenario 4**	Malawi	**All**	*	≥0.5	17,000	44%	19%	9%	0.87	1.12	1.33	1.52
		Females	15–34		10,000	48%	20%	11%	0.90	1.24	1.70	1.56
		Males	20–34		6,300	38%	17%	7%	0.84	0.96	0.99	1.44
	Mozambique	**All**	*	≥1.0	39,000	36%	16%	5%	1.67	1.95	2.25	2.84
		Females	15–34		24,000	39%	16%	5%	1.74	2.19	2.86	3.02
		Males	20–34		15,000	32%	17%	5%	1.57	1.65	1.77	2.63
	Zambia	**All**	*	≥1.0	19,000	43%	19%	4%	3.32	4.37	5.29	6.10
		Females	15–34		13,000	50%	21%	4%	3.64	5.10	7.80	7.28
		Males	20–34		6,700	34%	17%	4%	2.86	3.45	3.48	5.11

* Women aged 15–34 years and men aged 20–34 years.

Scenario 1: no disaggregation by age group or district.

Scenario 2: disaggregation by age group.

Scenario 3: disaggregation by district incidence.

Scenario 4: disaggregation by district incidence and high-incidence age group (females 15–34 years, men 20–34 years).

NA, not applicable; STI, sexually transmitted infection; STINP, sexually transmitted infection or non-regular partners.

### Scenario 2: New HIV infections by sex and age

In all 3 countries, more than 3 quarters of all new infections occurred among men and women between the age of 15 and 34 years (Table 2). For women, the age group of 15 to 34 years was most affected by HIV; for men, the most affected age groups were 20 to 34 years, likely because sexual debut is usually later than among women. In these age groups, the HIV incidence is higher for men and women than in older age groups (Table 2).

### Scenario 3: New HIV infections by district incidence

**Fig 1:**
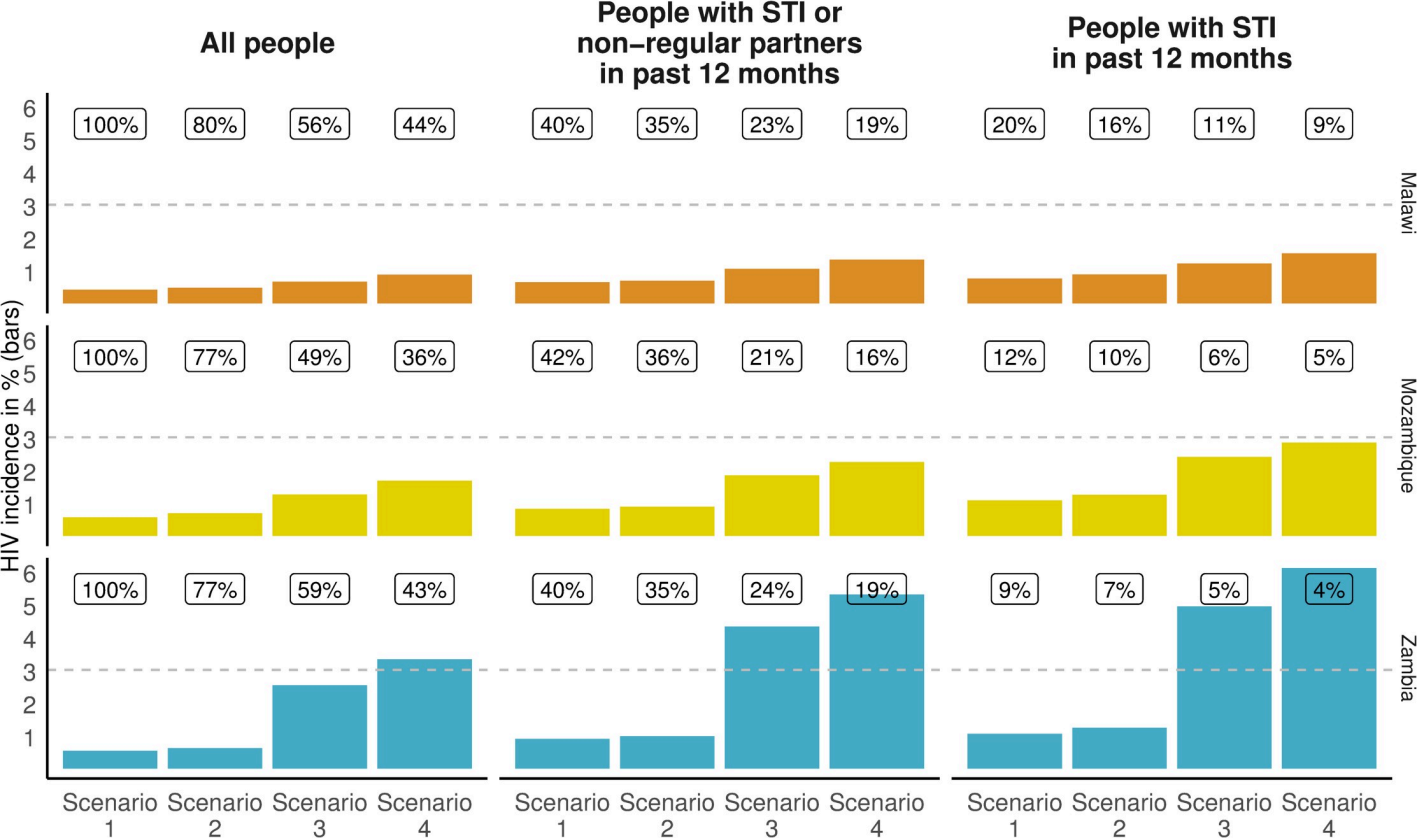
HIV incidence and proportion of new HIV infections potentially averted^⸸^ by scenario and priority population. Scenario 1: all people, scenario 2: people aged 15–34 years, scenario 3: people living in high-incidence districts, and scenario 4: women aged 15–34 years and men aged 20–34 years who live in high-incidence districts. STI, sexually transmitted infection. ^⸸^Proportion of new HIV infections that could be averted are shown as numbers in boxes.

In Malawi, not a single district had an incidence ≥1.0%, but 8 out of 28 districts had incidence rates ≥0.5%. Approximately 3.5 million people reside in these districts. In Mozambique, 62/159 districts had an incidence ≥1.0% where more than 4.2 million people live. In Zambia, the epidemic is concentrated on fewer districts (55/103) in which 3.2 million people live. The average HIV incidence in districts where around half of all HIV infections occur is 0.66% in Malawi, 1.26% in Mozambique, and 2.53% in Zambia. The HIV incidence among people in the category STINP in the past 12 months in these high-incidence districts was 1.05% in Malawi, 1.84% in Mozambique, and 4.32% in Zambia. Among people with STI in the past 12 months, the respective HIV incidence rates were 1.21% in Malawi, 2.40% in Mozambique, and 4.93% in Zambia (Fig 1).

### Scenario 4: New HIV infections by sex, age, and district

In Malawi, Mozambique, and Zambia, 44%, 36%, and 42% of all new infections occurred among women aged 15 to 34 years and men aged 20 to 34 years who lived in high-incidence districts. These proportions represent 17,000, 39,000, and 19,000 new HIV infections (Table 2). By selecting men and women in these age groups who lived in high-incidence districts and who had an STI in the past 12 months, the HIV incidence increased to 1.52% in Malawi, 2.84% in Mozambique, and 6.10% in Zambia (Table 2, Fig 1). In all 3 countries, the HIV incidence was higher among young women than among young males.

**Fig 2:**
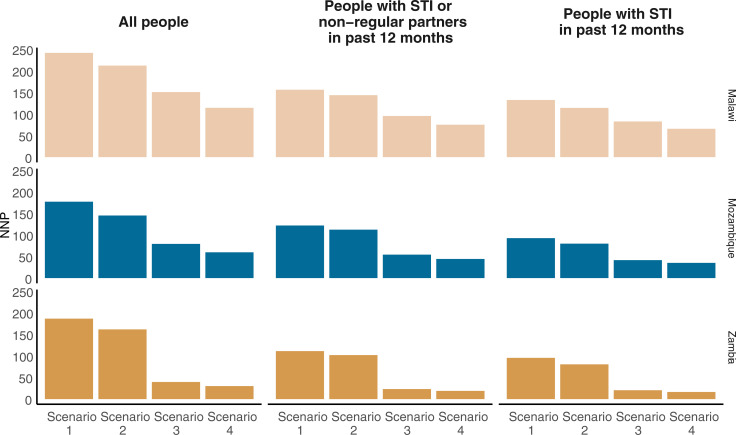
NNP by scenario and priority population. Scenario 1: all people, scenario 2: people aged 15–34 years, scenario 3: people living in high-incidence districts, and scenario 4: women aged 15–34 years and men aged 20–34 years who live in high-incidence districts. NNP, number of person-years on PrEP needed to prevent 1 HIV infection; PrEP, pre-exposure prophylaxis; STI, sexually transmitted infection.

In summary, in Malawi, there was no district, age group, or population with behavioural risk factors that had an average HIV incidence above the ≥3% PrEP threshold. In Mozambique, young women (aged 15 to 34 years) who lived in high-incidence districts and who had an STI in the past 12 months are above the 3% threshold for PrEP. Due to the high overall incidence in Zambia, different subpopulations could be prioritised for PrEP depending on resources. The most infections potentially averted could be obtained by encouraging PrEP use by all sexually active adults living in high-incidence districts. This would mean 805,000 person-years of PrEP being used and thereby potentially averting up to 26,000 new HIV infections (Fig 2). If PrEP were prioritised only to certain age groups (females 15 to 34 years and males 20 to 34 years), it would take 545,000 person-years of PrEP to potentially avert up to 19,000 infections. Focusing instead on only younger people who live in high-incidence districts and who were categorised as STINP would mean 165,000 person-years of PrEP to avert up to 8,700 new HIV infections. Focusing on younger people who had an STI in the past 12 months would mean that 28,000 person-years on PrEP would need to be taken in order to avert up to 1,700 new HIV infections. The proportion of infections potentially averted out of all new HIV infections with this focused approach would be very small (4%) and if a PrEP effectiveness of 50% is applied, would be only half of these infections (Table 3).

**Table 3:** Scenarios identifying priority populations with HIV incidence ≥3% and HIV infections that potentially could be averted.

	Malawi	Mozambique	Zambia				
Population for prioritisation	None						
Sex	-	Females	Both sexes	Females	Both sexes	Both sexes	Both sexes
Age group (years)	-	15–34	15+	15+	15/20–34	15/20–34	15/20–34
District incidence	-	≥1%	≥1%	≥1%	≥1%	≥1%	≥1%
Risk factor	-	STI	Sexually active	Sexually active	Sexually active	STINP	STI
Priority population size	0	99,000	805,000	360,000	545,000	165,000	28,000
HIV infections*	0	3,000	26,000	15,000	19,000	8,700	1,700
HIV incidence	-	3.0%	3.3%	4.1%	4.4%	5.3%	6.1%
NNP*** (100% efficacy)	**-**	**33.1**	**30.3**	**24.2**	**22.9**	**18.5**	**16.4**
HIV infections averted (50% effectiveness)** [uncertainty interval 25%–75%]	-	1,500 [750–2,250]	13,000 [6,500–19,500]	7,500 [3,750–11,250]	9,500 [4,750–14,250]	4,350 [2,180–6,530]	850 [430–1,280]

*Occurring in the priority population.

**HIV infections that could be averted if 50% [25%–75%] of all people in the priority population would initiate and adhere to PrEP.

^***^NNP, number of person-years on PrEP needed to prevent 1 HIV infection.

PrEP, pre-exposure prophylaxis.

## Discussion

Decisions on where and to whom to offer PrEP as a priority are complex and need to consider estimated numbers of individuals at risk of HIV, cost, equity, and other factors. Combining the geospatial mapping of HIV incidence with age, sex, and easily accessible markers of behavioural risk more precisely identified subpopulations with likely high HIV incidence which includes many of those who may benefit from PrEP and would theoretically inform a cost-effective way of delivering PrEP in Malawi, Mozambique, and Zambia. Our findings from Malawi, Mozambique, and Zambia showed that even in settings with high HIV prevalence, the numbers needed to prevent 1 infection are high, but decline quickly if approaches that use age, sex, and geospatial risk are considered in program planning. The average HIV incidence in districts where half of all infections occurred varied considerably (0.7%, 1.3%, and 4.9%, respectively) and increased when combined with sex, age, and STIs to 1.5%, 2.8%, and 6.1% in each country. In Malawi, no combination of district, sex, age, and risk group resulted in an incidence above the 3% incidence threshold suggested by WHO and UNAIDS as being potentially cost-effective for PrEP. Using this approach to PrEP prioritisation, the lowest numbers of person-years of PrEP needed to prevent 1 infection were 33.1 in Mozambique and 16.4 in Zambia.

Considering geography is critical in the prioritisation of PrEP services since HIV prevalence and incidence vary considerably in countries with generalised epidemics [20–22]. Our analysis showed that approximately half of new infections occurred in districts with overall HIV incidence rates ≥1.0% in Mozambique and Zambia and ≥0.5% in Malawi. Zambia was the only country which had 7 districts over the 3% incidence threshold. In all 3 countries, over 3 quarters of new infections occurred among men and women between the age of 15 and 34 years, with few in the 15 to 19 years age group. Using these sex- and age-specific data further identified subpopulations with higher HIV incidence; even so, Zambia remained the only country which had a 3% incidence in any subgroup; Mozambique reached nearly 2% for women. Therefore, at a population level, further prioritisation of PrEP services required the inclusion of individual sexual behaviour.

Focusing support at the individual level based on age, sex and risk behaviour is commonly used to offer PrEP in clinical settings [23–25] and often centres on a history of sex without condoms, partner HIV status, and STIs. Our analyses using the magnitude of the association between behavioural risk factors for HIV from survey data showed that having had an STI in the past 12 months and having an NP would serve as good markers of HIV “substantial risk” in districts where the background incidence of HIV was already moderately high. Combining these factors together led to the identification of multiple age-, sex-, and location-specific subpopulations that are likely to include most people who are at substantial risk in both Mozambique and Zambia. These estimates of PrEP “need” could be used as an upper bound of a PrEP target since offering PrEP outside these bounds would be expensive. In Malawi, no combination of district, sex, age, and risk group resulted in an incidence above 3%. However, in Malawi, and indeed in most countries, there will be individuals who may benefit from PrEP who were not considered in our approach. Focusing on key populations (sex workers, men who have sex with men, transgender people, prisoners, and people who use/inject drugs) or age- and sex-specific groups with known high HIV incidence and prevalence would prevent HIV infections [26]. As has been shown in Australia, the UK, and the US, where HIV prevalence is comparatively low and infections concentrated in key populations, specifically men who have sex with men, the greatest declines in new diagnoses among priority populations have been seen where PrEP has been used alongside expanded access and coverage of ART [27–29].

Finding a balance between impact and cost is a challenge in all areas of public health and particularly so for comparatively more expensive prevention interventions such as PrEP. In our scenarios, focusing on only a part of the total population, the numbers of person-years of PrEP to prevent 1 HIV infection ranged from 33 to 16 with the largest number of HIV infections potentially averted occurring if PrEP was offered to all sexually active adults living in high-incidence districts. Yet offering PrEP to such large numbers of people may be cost prohibitive in many settings. Conversely, while increasing the number of risk factors considered (geography, age, sex, and behaviour) for a focused approach increases the estimated HIV incidence and therefore the cost-effectiveness in the subpopulation being considered, it simultaneously decreases the number of infections potentially averted. Therefore, PrEP targets based on a very focused combination of factors may be too small to achieve greater public health impact. Such a target may however provide a pragmatic approach for early target setting during PrEP roll out at a national/subnational level, particularly as our findings show a larger proportion of new HIV infections occurred among people with an STI or NPs in the past 12 months. While focused approaches may assist policy makers in prioritising resources, from a societal perspective, efforts to increase awareness and demand for PrEP more broadly through positive messaging for anyone in need have the benefit of reducing stigma and barriers to access that may occur if PrEP is perceived to be only for certain age groups, locations, or key populations [30].

The prevalence of reported STIs across all 3 countries ranged from 3.8% to 13.6% and was highest in Malawi. As etiological diagnosis of STIs is not currently available in many low- and middle-income settings, and syndromic approaches are more commonly used, this is likely to have limitations. Nevertheless, assessing history of an STI is a useful simple screen indicating higher HIV risk. Identifying people with an STI could be more easily achieved in a clinical setting and integrating PrEP into existing services where some level of STI management is offered (e.g., sexual and reproductive health services); PrEP users are also noted to have high rates of STIs [31–33]. Since the estimate includes all individuals with these risk factors, some of whom will not present to clinical services and will be difficult to identify and offer PrEP to, these targets will be aspirational.

Although this multilevel approach is needed to prioritise resources for PrEP at a subnational level, a large number of new infections may occur outside geographically prioritised areas. Therefore, HIV prevention services and potentially PrEP may be needed in these areas. While population-level HIV incidence, sex and age distributions, and STI prevalence inform resource distribution, individual-level data on behaviours and recent history of STI could be used to prompt PrEP counselling at any service delivery site. This requires the integration of PrEP and HIV prevention into other health services such as sexual and reproductive health and antenatal and postnatal care. PrEP services can have wider benefits beyond preventing HIV infection such as bringing people who have not previously sought HIV testing and have self-identified as having higher HIV-risk into services. This could result in increased HIV testing, diagnosis, and linkage to treatment for those with HIV alongside access to PrEP for those at substantial risk. Increasing PrEP availability, uptake, and continuation by individuals at substantial risk is critical if population-level reductions in HIV infections are to be achieved. Recent adaptations, some prompted by the Coronavirus disease 2019 (COVID-19) pandemic restrictions, which simplify PrEP access such as community-based services and telemedicine, have been well received by PrEP users and have potential for the future [34–36].

Limitations of our approach are that we used modelled estimates of new HIV infections at a subnational level as it was the only way to estimate age-, sex-, and district-specific measures of new infections. Estimates of incidence in the HIVE model relied heavily on programmatic treatment data that had mixed quality; more recent modelling efforts have improved the review of input data and thus the district-level incidence estimates. The models were calibrated to 2016 data, and the DHS surveys were from the same period. As overall HIV incidence falls, the numbers needed to PrEP to prevent a new HIV infection will tend to rise.

We derived our risk estimates for behavioural risk factors from nationally but not subnationally representative HIV data and also from non-longitudinal data. We applied these to HIV incidence estimates but given that no longitudinal age- and sex-specific risk estimates for the effect of STI and STINP on HIV incidence were available, we considered the use of PRs from population-based cross-sectional data to be useful, nonetheless. Furthermore, the survey data we used to determine STI and NP prevalence were based on self-report and therefore were likely underestimated since STIs in women are often asymptomatic, and there may be social desirability bias associated with reporting NPs. Operationalising prioritisation approaches is likely to also use self-reporting in the medium term. As had been found in most sexual behaviour surveys, in the selected countries, considerably more men reported non-regular sexual partnerships in the past 12 months compared with women. Our estimate of PrEP need would therefore be conservative. We assumed that PrEP efficacy was 100% for this hypothetical PrEP target estimating exercise. Yet PrEP must first reach the intended population and then must be taken as prescribed to avert new infections. We therefore scaled back the number of HIV infections averted to allow for a 50% effectiveness. Both coverage of PrEP services and uptake and continuation on PrEP are currently low in all 3 countries, and therefore, our estimate of infections averted may be optimistic. Furthermore, not all people with risk factors for HIV need PrEP. Some may choose other prevention options, including consistent condom use.

We provide a pragmatic approach to allow estimates of the size, incidence, and impact of offering PrEP to subpopulations by combining geospatial HIV incidence estimates with publicly available population-based risk data. Analyses of this sort can be used to gain an understanding of who may benefit from PrEP in a general population setting and may help countries to consider appropriate numerical targets and population focus for PrEP programmes. This analysis could also be important for considerations for the introduction of new biomedical prevention options, such as long-acting PrEP. As more options for PrEP become available, their strategic introduction will be important. Using 3 countries as examples, we show that by prioritising risk factors, high-incidence subpopulations can be identified, although in Malawi, none reached the 3% incidence threshold. This analysis could provide a starting point for understanding PrEP need, prioritising resources, and focusing services for populations beyond key populations in high-HIV-burden countries which are considering or implementing PrEP.

## Disclaimer

The contents in this article are the sole responsibility of the authors and do not necessarily reflect the views of WHO, USAID, PEPFAR, or the United States Government. DC was previously employed by USAID for a period of time during which this study was conducted.

## Supporting information

S1 Text Variables for the calculation of new HIV infections, HIV incidence, and NNP. NNP, number of person-years on PrEP needed to prevent 1 HIV infection.
(DOCX)

S1 Table (A) Number of new HIV infections, incidence, prevalence, and proportion of people with an STI or NPs in the past 12 months in Malawi, Mozambique, and Zambia. (B) PRs of HIV prevalence among people with and without “STI or NP in the past 12 months.” NP, non-regular sexual partner; PR, prevalence ratio; STI, sexually transmitted infection.
(DOCX)

S2 Table RECORD checklist. (DOCX)

## Abbreviations

AIS: AIDS Indicator Surveys
ART: antiretroviral therapy
COVID-19: Coronavirus disease 2019
DHS: Demographic and Health Surveys
NP: non-regular sexual partner
NNP: number of person-years on PrEP needed to prevent 1 HIV infection
PLHIV: people living with HIV
PR: prevalence ratio
PrEP: pre-exposure prophylaxis
STI: sexually transmitted infection
STINP: sexually transmitted infection or non-regular partners
TDF: tenofovir disoproxil fumarate
UNAIDS: Joint United Nations Programme on HIV/AIDS
