# Supplementary material for: Estimating HIV pre-exposure prophylaxis need and impact in Malawi, Mozambique and Zambia: A geospatial and risk-based analysis
Source: PLoS Med. 2021 Jan 11;18(1):e1003482. doi: 10.1371/journal.pmed.1003482 (PMC7799816; doi:10.1371/journal.pmed.1003482)
Supplement: S1 Text — NNP, number of person-years on PrEP needed to prevent 1 HIV infection. (DOCX) [file pmed.1003482.s001.docx]

**S1 Text**

**Variables for the calculation of new HIV infections, HIV incidence and NNP**

r being the risk factor

STI if people reported having had an STI in the past 12 months

r

STINP if people reported having had an STI and/or a

concurrent partners in the last 12 months

a being the age group

1 if 15–19 years,

2 if 20–24 years,

3 if 25–29 years,

a

4 if 30–34 years,

5 if 35–39 years,

6 if 40+ years,

and s being sex.

m if male,

s

f if female
