# Supplementary material for: Estimating HIV pre-exposure prophylaxis need and impact in Malawi, Mozambique and Zambia: A geospatial and risk-based analysis
Source: PLoS Med. 2021 Jan 11;18(1):e1003482. doi: 10.1371/journal.pmed.1003482 (PMC7799816; doi:10.1371/journal.pmed.1003482)
Supplement: S1 Table — (A) Number of new HIV infections, incidence, prevalence, and proportion of people with an STI or NPs in the past 12 months in Malawi, Mozambique, and Zambia. (B) PRs of HIV prevalence among people with and without “STI or NP in the past 12 months.” NP, non-regular sexual partner; PR, prevalence ratio; STI, sexually transmitted infection. (DOCX) [file pmed.1003482.s002.docx]

**S1 Table A. Prevalence ratios of HIV prevalence among people with and without STI in the past 12 months**

| **Sex** | **Age group** | **STI among PLHIV** | **STI among HIV negative individuals** | **HIV prevalence among people with STI*** | **HIV prevalence among**  **people with**  **no STI*** | **Prevalence Ratio**  **[95% CI]** |
| --- | --- | --- | --- | --- | --- | --- |
| Females | 15–24 | 92 / 707 | 663 / 7940 | 13.1% | 8.4% | 1.56 [1.27–1.92] |
|  | 25–29 | 83 / 391 | 553 / 4212 | 21.1% | 13.1% | 1.61 [1.28–2.02] |
|  | 30–34 | 95 / 329 | 700 / 3719 | 28.9% | 18.8% | 1.54 [1.23–1.92] |
|  | 35–39 | 82 / 269 | 684 / 3143 | 30.5% | 21.8% | 1.40 [1.10–1.80] |
|  | 40+ | 108 / 315 | 774 / 4032 | 34.2% | 19.2% | 1.78 [1.43–2.22] |
| Males | 15–29 | 82 / 874 | 525 / 9650 | 9.3% | 5.4% | 1.72 [1.39–2.13] |
|  | 30–34 | 55 / 243 | 369 / 3021 | 22.6% | 12.2% | 1.85 [1.39–2.46] |
|  | 35–39 | 53 / 211 | 404 / 2645 | 25.1% | 15.3% | 1.64 [1.22–2.21] |
|  | 40+ | 67 / 212 | 672 / 3809 | 31.5% | 17.7% | 1.78 [1.35–2.36] |

* HIV prevalence among males and females who ever had sex

**S1Table B. Prevalence ratios of HIV prevalence among people with and without ‘*STI and/or non-regular partner in the past 12 months’***

| **Sex** | **Age group** | **STINP among PLHIV** | **STINP among HIV negative individuals** | **HIV prevalence among people with STINP*** | **HIV prevalence among**  **people with**  **no STINP*** | **Prevalence Ratio**  **[95% CI]** |
| --- | --- | --- | --- | --- | --- | --- |
| Females | 15–19 | 111 / 1506 | 94 / 1950 | 7.3% | 4.8% | 1.53 [1.34–1.75] |
|  | 20–24 | 216 / 1344 | 336 / 3849 | 16.0% | 8.7% | 1.84 [1.64–2.07] |
|  | 25–29 | 200 / 862 | 436 / 3742 | 23.2% | 11.7% | 1.99 [1.74–2.27] |
|  | 30–34 | 246 / 702 | 549 / 3346 | 35.0% | 16.4% | 2.14 [1.87–2.44] |
|  | 35–39 | 219 / 587 | 546 / 2824 | 37.3% | 19.3% | 1.93 [1.67–2.23] |
|  | 40+ | 216 / 598 | 666 / 3750 | 36.1% | 17.8% | 2.03 [1.75–2.36] |
| Males | 15–24 | 154 / 4518 | 119 / 2590 | 3.4% | 4.6% | 0.74 [0.67–0.82] |
|  | 25–29 | 176 / 1342 | 158 / 2075 | 13.1% | 7.6% | 1.72 [1.54–1.92] |
|  | 30–34 | 170 / 906 | 253 / 2356 | 18.8% | 10.8% | 1.75 [1.53–1.99] |
|  | 35–39 | 140 / 671 | 316 / 2184 | 20.9% | 14.5% | 1.44 [1.24–1.69] |
|  | 40+ | 152 / 663 | 587 / 3358 | 23.0% | 17.5% | 1.31 [1.12–1.55] |

* HIV prevalence among males and females who ever had sex
